# Wetland biomass inversion and space differentiation: A case study of the Yellow River Delta Nature Reserve

**DOI:** 10.1371/journal.pone.0210774

**Published:** 2019-02-05

**Authors:** Mei Han, Bin Pan, Yu Bin Liu, Hao Zhe Yu, Yan Rong Liu

**Affiliations:** School of Geography and Environment, Shandong Normal University, Jinan, China; Assam University, INDIA

## Abstract

With wetlands categorized as one of the three major ecosystems, the study of wetland health has global environmental implications. Multiple regression models were employed to establish relationships between Landsat-8 images, vegetation indices and field measured biomass in the Yellow River Delta Nature Reserve. These models were then used to estimate the spatial distribution of wetland vegetative biomass. The relationships between wetland vegetative biomass and soil factors (organic matter, nitrogen, phosphorus, potassium, water soluble salt, pH and moisture) were modeled. We were able to achieve higher correlations and improved model fits using vegetative indices and spectral bands 1–5 as independent variables. Several important soil factors were isolated, including soil moisture and salt concentrations, which affect wetland biomass spatial distributions. Overall, wetland biomass decreased from land to the ocean and from the river courses outward.

## Introduction

Wetlands, forests and oceans comprise the three most productive ecosystems on Earth. Although wetlands account for a relatively small proportion of total productivity, their contribution is nonetheless irreplaceable. Wetlands have therefore been described as "the kidney of the Earth" and "the species gene pool". The Ramsar Convention classifies wetlands as natural areas of marsh, peatland and forested swamps[1]. Wetland vegetation biomass incorporates the total mass of living vegetation within a given area, and this important index measures vegetation productivity and describes the circulation of material, energy flow processes, ecological function and ecosystem health within a wetland ecosystem[2–5].

The study of biomass can be traced back to the earliest research on forest litter and wood weight data, as conducted by the German scholar Ebemeyer in 1876, which was limited to specific tree species[6] Rapid development of forest biomass research occurred during the 20^th^ century at all spatial scales, from regional evaluation of biomass to the interactions between woodland soil microbes, fungi and nutrients.

Chinese scholars began studying forest biomass in the 1970s [7] The Sixth National Forest Resources Inventory mapped the distribution of vegetation at a scale of 1:1,000,000, thereby allowing the calculation of the spatial distribution of net primary productivity (NPP) based on Moderate Resolution Imaging Spectroradiometer (MODIS) inversion. Based on the determination of the aboveground and underground biomasses of plant communities in an alpine meadow of the Qinghai Tibetan Plateau over a span of four years [8] Yang et al. explored the correlations of carbon, nitrogen (N) and phosphorus (P) inputs with aboveground biomass[9]. Through the analysis of microbial respiration and micro-environmental factors of soil in the Shanghai Jiuduansha Wetland National Nature Reserve, Jia et al. found that microbial diversity and soil microbial biomass were the most significant factors affecting soil microbial respiration[10].

Since the 1990s, researchers have increasingly focused on studying wetland biomass using remote sensing and the accompanying statistical estimation models[11–14]. Valk et al. found that seasonal changes in water level and temperature affect all forms of vegetative biomass in the India Keoladeo National Park[15]. By using hyper spectral remote sensing, Mirik et al. developed an accurate model at 1 m resolution for modeling the vegetative biomass of pastures in the Yellowstone National Park[16]. Austin et al.[17] used non-metric multidimensional scaling on seven vegetative biomass types, thereby establishing that wetland hydrological conditions are the primary factors affecting vegetative biomass in a wetland in southeast Idaho. Fuller and Feng [18,19] used 10 years of remote sensing images and the normalized difference vegetation index (NDVI) to analyze the changes in vegetation structure for a wetland in the South Florida peninsula, with the results suggesting that increased saline intrusion associated with sea-level rise continues to reduce the photosynthetic biomass within freshwater and oligohaline marsh communities of the southeastern Everglades. Using data collected from 1998–2005 for a wetland in southern California, Daniels et al. [20] identified a strong correlation between *Phragmites australis* dry weight, density and aboveground biomass. Using multispectral remote sensing and non-parameter modeling, Güneralp and Filippi [21] estimated the biomasses of floodplain areas using stochastic gradient (SGB) and multivariate adaptive regression (SARS) analyses to obtain a more reliable result, which paved the way for research on regional and global scales. Using enhanced thematic mapper (ETM) and sampling data for Poyang Lake, Li and Liu [22] estimated the lake’s biomass using linear fitting and Albert projection.

By comparing HJ-1A charge-coupled device (CCD) satellite remote sensing and field measurement data for the Yellow River Delta (YRD), Fu et al. [23] was able to accurately estimate the fresh weight of *Suaeda heteroptera* Kitag., thereby providing a more efficient method to monitor and evaluate wetland biomass and ecological function. Using the YDR Nature Reserve as an experimental area, Gao et al. [24] achieved high-precision estimations of wetland vegetative biomass using multivariable linear regression modeling (MLRM).

At present, two remote sensing (RS)-based methods exist to estimate wetland biomass: optical and radar. Because RS is economical, readily available and easy to process, optical RS has been widely used. Radar RS can be used in a wider range of conditions, including during day or night in all types of weather, and has better penetration capabilities, which is advantageous when estimating vegetation biomass, particularly spectral bands L and C applied to forest land and low biomass vegetation wetlands, respectively. However, the factors most influencing wetland biomass can be divided into two categories: environmental factors and gene/phenotype factors. The former mainly includes temperature, moisture, light and soil properties, whereas the latter includes the number of species, species uniformity and spatial distribution, plant height, physiological regulation and carbon dioxide fixation. The dominant factors limiting biomass depends on specific circumstances.

In summary, research on wetland vegetative biomass has made the following progress: (1) Research methods have progressed from field sampling, drying and weighing to remote sensing and simulation modeling. Traditional methods of surveying wetland biomass have obvious limitations, including the manpower required and the limited spatial extend possible, whereas modern remote sensing technology can not only overcome these shortcomings, but can also objectively estimate wetland biomass through a full range, at a large spatial scale and through multi-angle remote sensing images; (2) The relationships between wetland vegetative biomass, hydrological conditions, climate and other factors are increasingly well understood. Wetland vegetation is an important part of the wetland ecosystem, and environmental factors are closely related to wetland biomass. The study of these factors can result in an improved quantitative understanding of the specific factors most influencing wetland vegetation biomass, thereby providing a scientific basis for the protection and restoration of wetland ecosystems. The majority of past studies explored the relationships of wetland biomass with hydrology and climate though outdoor sampling, monitoring and indoor testing, physical/chemical analysis and establishing regression models; (3) Biomass inversion models are now effective methods to study wetland biomass. By combining the traditional collection of sample data with the analysis of remote sensing images, wetland vegetation biomass can be more accurately estimated[25–27].

These advances have made it possible to study the relationship between vegetative biomass and environmental factors. Therefore, the present study used Landsat-8 imaging of the YDR Nature Reserve to evaluate the effect of soil organic matter (OM), total nitrogen, total phosphorus, total potassium (K), water soluble salt and soil reaction (pH) on wetland biomass to explore the correlation between the NDVI, difference vegetation index (DVI), ratio vegetation index (RVI) and spectral bands 1–6 with vegetative biomass. To provide scientific guidance and a reference for wetland biodiversity conservation and wetland restoration, we calculated total biomass and generated a biomass distribution map for the study wetland, following which we discuss how vegetative biomass is influenced by species diversity using the Simpson Diversity Index (SDI), Richness Index (RI) and Shannon Index. Finally, we analyzed the spatial distribution of biomass to explore the relationship between biomass and soil parameters.

## Materials and methods

### Study location

**Fig 1:**
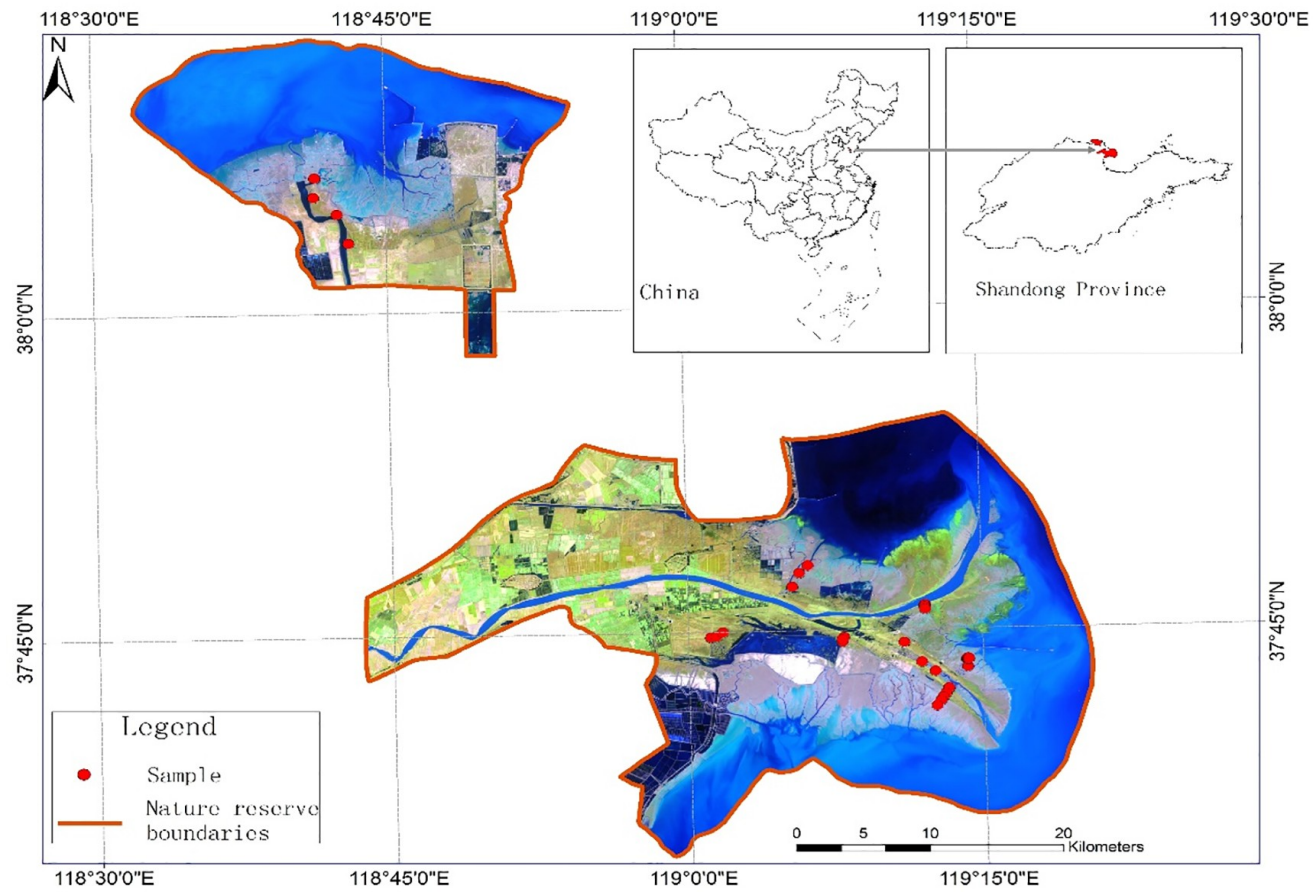
The Yellow River Delta Nature Reserve and samples taken for the study of wetland biomass.

Two sites within the YDR Nature Reserve were chosen for the present study. The YDR Nature Reserve forms part of the National Nature Reserve Administration of ShanDong Province, China. The chosen sites are located at the mouth of the Yellow River, one to the west of Laizhou Bay and the other to the north of Bohai Sea. The sites are separated from the Laodong Peninsula by the sea (37°35′–38°12′ N, 118°33′–119°20′ E). One site is located in the current Yellow River Estuary and covers an area of 104,500 ha, whereas the other is situated at the position of the Yellow River’s previous estuary before the river changed course in 1976, and covers an area of 48,500 ha (Fig 1). The landforms of the YDR are complex, and can generally be divided into dryland, tidal flats and the subtidal zone. The study area has a semi-humid, monsoon climate. The annual average temperature is 11.9°C and annual average rainfall is 592.2 mm.

To begin with, we didn’t have any relevant permit numbers, but we have the name of permitting agency, that is the Yellow River National Nature Reserve Administration of ShanDong Province, China, which has the power to allow us enter the field and extract the soil samples. In addition, this research was funded by National Natural Science Foundation of China (41371517) and the Shandong Science and Technology Research Program (2013GSF11706), both of them are grant numbers. Moreover, we submitted the field investigation plan to the Yellow River National Nature Reserve Administration of ShanDong Province, China. Then, the Director of Scientific Research Department (Zhu Shuyu) and the Chairman of the Union (Lv juanzhang) verified and approved. After we obtained the permissions, we could extract the soil samples at the YDR Nature Reserve. And all the soil samples are extracted from the public land, which belongs to the Yellow River National Nature Reserve Administration of ShanDong Province, China.

Also, there are numerous endangered birds in the Yellow River National Nature Reserve. 7 species of birds are the first-grade State protection, such as *rus japonensis*, *Otis tarda dows*, *Haliaeetus abbicuio albicilla*, *Ciconia bovciana* and *Mergus squamatus*,etc. Also, there is a national secondary protected plant: Glycine soja Sieb, et Zucc.

*The locations were not affect the* threatened species, the reasons are as follows:

1. After careful consideration, all the locations were kept away from the species habitat and there are no endangered species in the sample range.
2. Soil samples are collected in the field every October. During this period, some endangered species are not in the reserve because they come to the reserve only in November and December. Such as *rus japonensis*, *Otis tarda dow*, *Ciconia bovciana*.
3. The soil depth of all samples is 10 cm, and the weight is 500 g, which is very small and has no effect on the endangered species environment.
4. All sampling procedures were supervised by Director Zhu Shuyu of the Scientific Research Department of the National Nature Reserve Administration of the Yellow River Delta, Shandong Province, China.

### Sample collection

Field samples (n = 43) were collected in August 2014 during the peak growth time of meadow and marsh biomass to identify the wetland growth status and obtain the highest correlation with remote sensing data. At each sampling location (Fig 1), a square quadrat (1 m × 1 m) made of polyethylene pipe was randomly thrown and the geographic coordinates of the center of the square were recorded by means of a handheld global positioning system (GPS) receiver. In addition, biomass coverage, vegetation type and average vegetation height within the square were recorded. Fresh weight of the above-ground biomass was documented and one-third of the above-ground biomass was sealed in plastic bags. At each sampling location, a topsoil sample (0–10 cm) was collected and placed in a plastic bag.

### Soil and biomass analysis

Biomass samples were initially oven-dried -at 80 °C for 8 h. They were further dried in a drying oven for 4 h and then the biomass dry weight was determined. Soil moisture was measured gravimetrically by means of a convection oven (105 °C). Furthermore, soil organic matter (OM) was determined through potassium dichromate titration [28] and soil pH was estimated in a 1:2 soil/water solution using a pH electrode. Nitrogen (N) concentration was determined using the Kjeldahl method and available phosphorus (P) was extracted using sodium bicarbonate and measured using photo-spectroscopy [29]. The amounts of water-soluble-salts were measured using filter evaporation.

### Remote sensing image extraction and processing

The remote sensing images were captured using the Landsat-8 Operational Land Imager (OLI) on 20 July 2014. Since the study area shows relatively minor variations in topography, the remote sensing images were processed using radiometric calibration, fast line-of-sight atmospheric analysis of spectral hypercubes (FLAASH) atmospheric correction and irregular image cutting[30]. Remote sensing data extraction was based on the reflectance of each band and the vegetation indices. Among the 11 spectral bands of the images, spectral bands 1–6 and the NDVI, DVI and RVI indices were selected due to their accuracy in imaging of coastal biomass.

### Biomass modeling

The RS retrieval model was established using the relationship between the vegetation index and biomass. A simple linear model could not accurately estimate biomass due to the influence of land use types, soil type and soil properties. Thus, before establishing the regression model, the influences of land use types, such as basic farmland, were removed from the vegetation coverage area to calibrate the results.

Using the Statistical Package for the Social Sciences (SPSS) software package, correlations between each remote sensing factor and vegetative biomass were calculated. The type of model chosen for each index was dependent upon the coefficient of determination. The highest correlation coefficient was then used to select the regression model from among the linear regression, nonlinear regression and multiple linear regression models.

Multi-dimensional linear regression modeling is superior to other evaluation models when eight factors (NDVI, DVI, RVI and spectral bands 1–5) rather than nine factors (by adding spectral band 6), are used as independent variables, and the coefficient of determination of *R*^*2*^ = 0.69 was the highest achieved.

The eight factors of the multivariate linear regression model were as follows:

Y=1335.203a+24279.102b-124.008c+32189.511d-32877.829e+3769.278f+16212.176g-18279.133h-62.5 (1)

where *a* is NDVI, *b* is DVI, *c* is RVI, *d* is band 1, *e* is band 2, *f* is band 3, *g* is band 4 and *h* is band 5.

The calculation of the average residual error coefficient was as follows:

$$Y\;=\;\frac{\sum_{i\;=\;1}^{n}\left|\frac{B_{i}-B_{i}}{B_{i}}\right|}{n}\times 100\%$$ (2)

Where *B* is the measured value, $\hat{B}$ is predicted, *B*_*i*_ is the measured biomass dry weight of the *i*th sample, ${\hat{B}}_{{}^{{}_{i}}}$ is the predicted biomass dry weight of the *i*-th sample and *n* is the sample population.

Because of the low precision of linear models, only multiple linear regression models were established. The prediction accuracy of the model was tested by retaining ten samples in the model. Average residual error coefficient has a negative relationship with predictive alignment of the model; therefore, an average residual coefficient of 0.09 and predicted inosculation of 85.85% indicates that the model precision is higher, and that the model can be used to simulate biomass in this area (Table 1).

**Table 1:** Tests for accuracy of the biomass multivariable linear regression models.

Number	1	2	3	4	5	6	7	8	9	10	Comment
**measured value**	579.34	680.58	314.98	650.21	703.56	420.87	450.78	802.97	920.34	440.52	**model precision** **Goodness-of-prediction** 85.85% **Mean residual coefficient** 0.09
**predicted value**	611.56	531.35	379.66	637.92	687.50	494.84	416.94	779.35	1,018.12	476.90	
**residual error**	−32.22	149.23	−64.68	12.29	16.06	−73.97	33.84	23.62	−97.78	−36.38	

## Results and discussion

### Vegetative biomass of the study area

The multiple linear regression models were then input to the ArcGIS grid calculator to generate a map showing the spatial distribution of vegetative biomass dry weight divided into five classes, as according to natural break-point classification (Table 2).

**Table 2:** Wetland classification and extent in the Yellow River Delta Nature Reserve based on dry weight of biomass.

Classification	Dry wt (g m^−2^)	Area (km^2^)	Extent (%)
1	< 319	401.2	65.2
2	319–502	106.6	17.3
3	502–711	74.0	12.0
4	711–1,064	28.5	4.6
5	> 1,064	5.3	0.9

**Fig 2:**
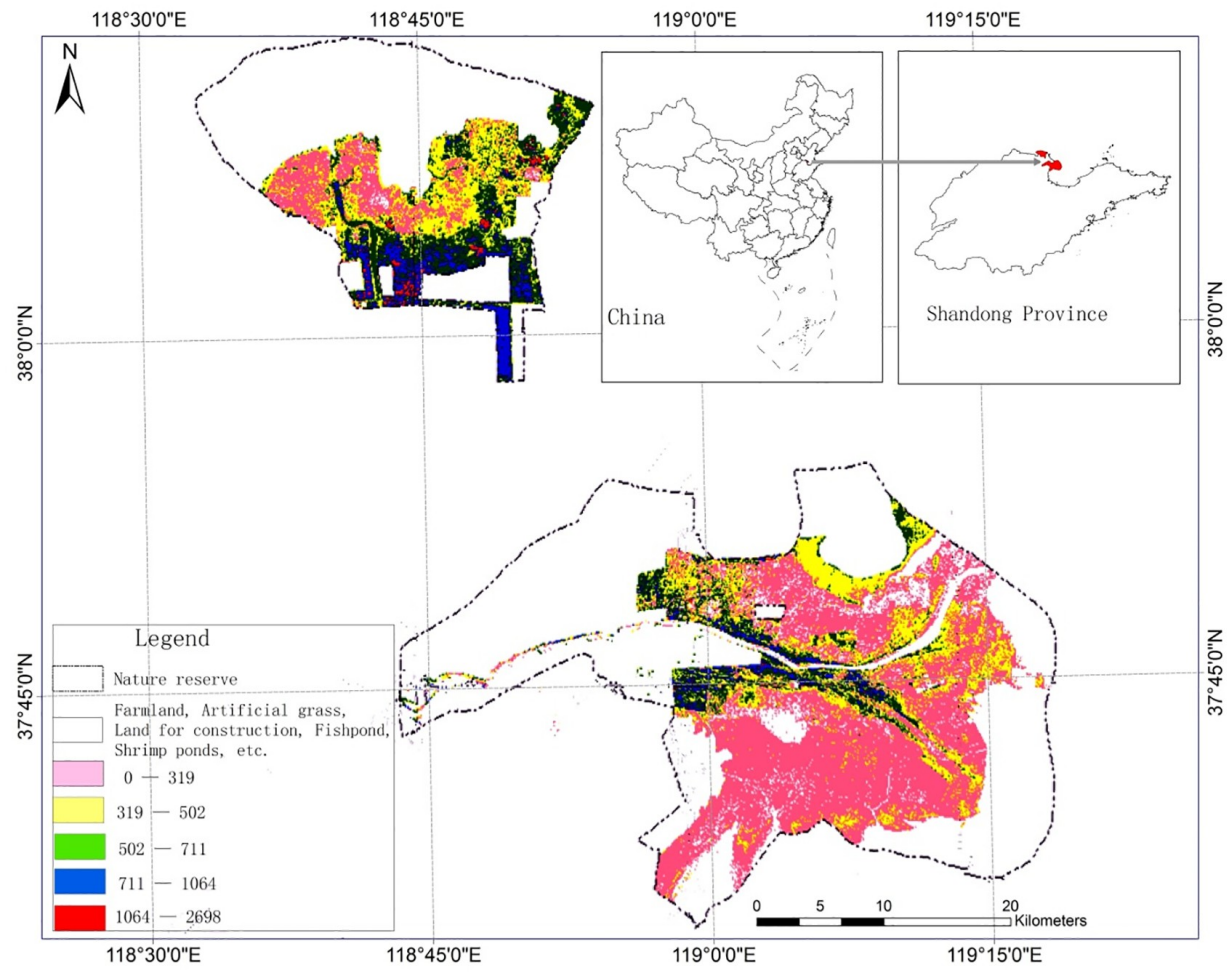
Spatial distribution of biomass density in the Yellow River Delta Nature Reserve.

The total estimated marsh and meadow biomass dry weight in the study area was 299,986 kg. Overall, an exponential inverse relationship between area extent and biomass quantity was evident (Table 2; Fig 2). Most of the land (65.2%) fell within Class 1 with the lowest biomass, followed by Class 2 (17.3%), Class 3 (12.0%), Class 4 (4.6%) and Class 5 (0.9%). The areas with the most biomass were located at the edge of an artificial woodland and farmland. Class 1 biomass was primarily distributed in areas with poor water quality and soil salinity.

### Spatial distribution of vegetative biomass

Vegetative biomass decreased with distance from the Yellow River and increased with distance from the sea. For the Yiqianer Station, vegetative biomass dry weight generally decreased from southeast to northwest (Fig 2). Areas of high biomass were located in the southern shrub swamps and marshes further from the coast and within wetland restoration areas where biomass growth is more influenced by human activities. Low biomass areas were located in the northern coastal muddy beaches and the ecological conservation areas. For the site of the previous river course, vegetative biomass dry weight decreased with distance from the riverbanks.

At Dawenliu Station, the course of the Yellow River from 1976–1996 and along the current river course, vegetative biomass was found to be dense (Fig 2); however, the density of the former river course was found to be significantly higher than that of the latter river course. During the cut off in 1976–1996, the river deposits were mature and the groundwater level was relatively high. However, the latter course which formed after 1996 remains in an early stage of development and the groundwater level is lower. Furthermore, higher salt concentrations occur near the coast and soil moisture is higher near the current course of the Yellow River.

### Effect of phragmites australis height on vegetative biomass

Feng and Zhao [19]found that the height of *Phragmites australis* (*P*. *australis*) in the YRD is directly proportional to water depth. To obtain more CO_2_ during photosynthesis, the height of vegetation will increase relative to water depth; therefore, *P*. *australis* height can reflect water depth. As a result, the related features between *P*. *australis* height and biomass can indirectly reflect the relationship between water depth and *P*. *australis* biomass.

The correlation coefficients between plant height and *P*. *australis* fresh and dry weights are 0.48 and 0.78, respectively, whereas the same correlation coefficients for *Suaeda salsa* (*S*. *salsa)*, a species of seepweed, are 0.28 and 0.19, respectively.

### Effect of soil factors on vegetative biomass

The dry weight of *P*. *australis* was found to be negatively correlated with soil water soluble salt and soil pH, indicating that these factors limit *P*. *australis* growth (Table 3). *P*. *australis* dry weight was positively correlated with P, OM and N, with the strength of the correlation from highest to lowest in that order, although soil moisture was found to have the highest positive correlations with P, OM and N. Since the YRD Nature Reserve is influenced by erosion and does not receive anthropological nutrient inputs, the soil layers are uniformly thin with little OM accumulation and low N and P concentrations. Therefore, N and P concentrations were not found to have a discernable correlation with *P*. *australis* dry weight. On the other hand, soil moisture and salt concentrations were highly correlated with *P*. *australis* dry weight. This illustrates that *P*. *australis* is well adapted to grow in weak alkaline soil environments.

**Table 3:** Correlation coefficients of two plant species and soil properties in the Yellow River Delta Nature Reserve wetlands.

	OM	Available N	Available P	Water-Soluble Salt	pH	Moisture%
*Phragmites australis*	0.087	0.127	0.023	−0.159	−0.159	0.387
*Suaeda salsa*	0.011	0.013	0.23	−0.013	0.069	−0.314

Abbreviations: organic matter (OM); nitrogen (N); phosphorus (P)

The dry weight of *S*. *salsa* was positively correlated with soil OM, N, P and pH and negatively correlated with water soluble salt and soil moisture. Since *S*. *salsa* is a halophyte and is adapted to alkaline soil, positive correlations between *S*. *salsa* dry weight and pH and soil moisture were evident. Soil water soluble salt concentrations are relatively high in many places near the coastal areas. Therefore, it is reasonable to assume a higher abundance of *S*. *salsa* would be found in regions with high concentrations of water soluble salt. However, in reality, since high soil moisture would also be found in areas with high water soluble salt concentration, the density and overall biomass of *S*. *salsa* is low.

Overall, the dry weights of *P*. *australis* and *S*. *salsa* were predominantly controlled by moisture, i.e., soil moisture was positively and negatively correlated with the growths of *P*. *australis* and *S*. *salsa*, respectively.

### Effect of species diversity on vegetative biomass

The Simpson Diversity Index (SDI), Richness Index (RI) and Shannon Index (SI) are commonly used to classify species diversity. These diversity indices were positively correlated with wet and dry vegetation weight (Table 4), with dry weight data showing higher correlation coefficients than fresh weight data. SI had the highest correlation with biomass weight, followed by SDI and then RI. Although SI accounts for the number of species, each species has equal weight. When the number of species and species distribution have little diversity, SI can accurately classify vegetative biomass.

**Table 4:** Correlation coefficients showing the relationship between Simpson’s diversity Index (SDI), Shannon’s Index (SI) and Richness Index (RI) with biomass in the Yellow River Delta Nature Reserve wetlands.

	SDI	SI	RI
Fresh wt.	0.318*	0.335*	0.230
Dry wt.	0.417**	0.449**	0.293

* significant at *p* ≤ 0.1 levels;

** significant at *p* ≤ 0.05.

**Fig 3:**
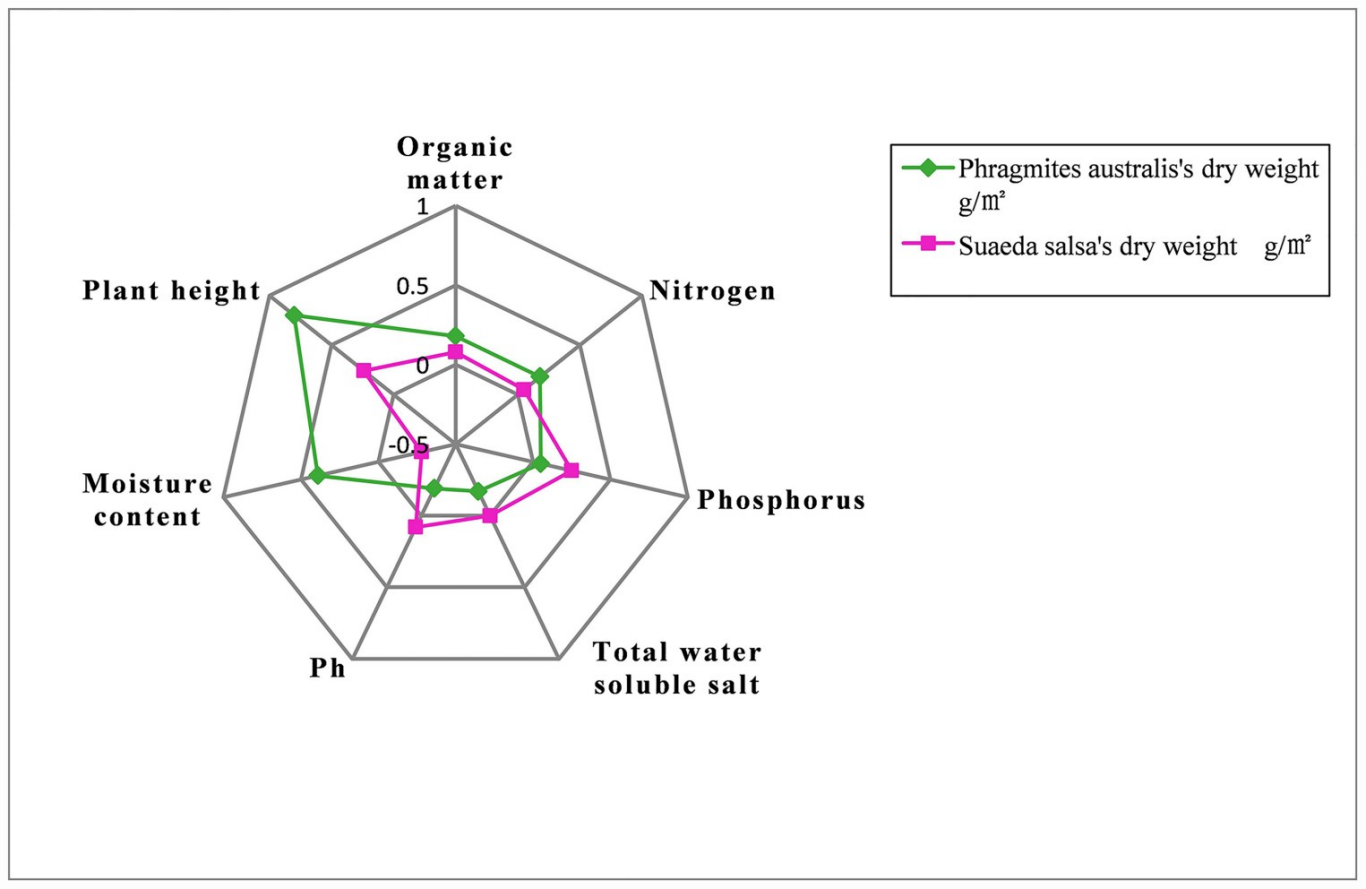
Radial map of the correlation coefficient of two vegetative species and selected environmental factors in the Yellow River Delta Nature Reserve wetlands.

Using these techniques, the spatial distribution and correlations of several driving factors of wetland vegetative biomass in the YRD Nature Reserve was explored. *Zeng* et al. [31] showed that biomass varies with elevation; however, elevation changes were not accounted for since changes in topography of the study area are minor. Among environmental factors, *P*. *australis* height had the greatest effect on *P*. *australis* biomass and there was no significant effect of OM and P. Soil water had the greatest effect on *S*. *salsa* biomass and OM had no significant effect (Fig 3). This shows that plant height and soil moisture are the key factors controlling *P*. *australis* and *S*. *salsa* growth, respectively. Although *P*. *australis* is well adapted to saturated conditions, *S*. *salsa* is less tolerant of salinity. The present study illustrated that not only the number of species, but also the spatial distribution of species is important for robust restoration and protection of biomass in the YRD.

## Conclusion

Using improved analysis techniques, stronger correlations between biomass dry weight and remote sensing data were obtained. Due to the unique hydrological relationship between soil and plants in wetlands, the actual biomass weight of perennial aquatic plants such as *P*. *australis* is lower than what field sampling can accurately reflect. Therefore, drying in the laboratory is a more accurate reflection of vegetative biomass in wetlands.

By employing multiple regression models, we were able to achieve stronger correlations and higher model fits with DVI, RVI, NDVI and spectral bands 1–5 as independent variables. The spatial distribution of vegetation dry weight was significantly correlated with various environmental factors, among which *P*. *australis* height had the greatest effect on *P*. *australis* biomass and P. Soil water had the greatest effect on *S*. *salsa* biomass, which illustrates that plant height and soil moisture are the key factors controlling *P*. *australis* and *S*. *salsa* growth, respectively, whereas both factors were positively correlated with vegetative biomass. At the same time, species diversity had a significant effect on biomass dry weight, thereby illustrating that the number of species and the evenness of the spatial distribution of species affect vegetative biomass. Thus, to promote the restoration and growth of biomass in natural areas, it is necessary to control soil moisture and salt concentrations to increase species distribution and biomass density.
